# Sustainable Implementation of Physician-Pharmacist Collaborative Clinics for Diabetes Management in Primary Healthcare Centers: A Qualitative Study

**DOI:** 10.1007/s44197-024-00244-2

**Published:** 2024-05-23

**Authors:** Jie Xiao, Shuting Huang, Qing Wang, Shenglan Tan, Lei Chen, Haiyan Yuan, Daxiong Xiang, Bikui Zhang, Xia Li, Yan Guo, Haiying Huang, Qun Li, Yaqi Liao, Yuhan Tan, Yining Cheng, Hao Lu, Ping Xu

**Affiliations:** 1 Department of Pharmacy, The Second Xiangya Hospital, Central South University, Changsha, CN China; 2 Institute of Clinical Pharmacy, The Second Xiangya Hospital, Central South University, Changsha, CN China; 3 Department of Endocrine, The Second Xiangya Hospital, Central South University, Changsha, CN China; 4 Department of Pharmacy, Taoyuan People’s Hospital, Changde, CN China; 5 Department of Pharmacy, The People’s Hospital of Liuyang, Changsha, CN China; 6 Department of Pharmacy, The Second People’s Hospital of Huaihua, Huaihua, CN China; 7 Intemed Hospital Management & Development (Beijing) Centre, Beijing, CN China

**Keywords:** Diabetes mellitus, Pharmacist, Primary healthcare, Disease management, Sustainable development, Implementation science

## Abstract

**Background:**

Although physician-pharmacist collaborative clinics for diabetes management have been shown to be effective and cost-effective worldwide, there is limited understanding of the factors that influence their sustainable implementation. This study aims to identify the associated factors and provide sustainability strategy to better implement physician-pharmacist collaborative clinics for diabetes management in primary healthcare centers in China.

**Methods:**

A sample of 43 participants were participated in face-to-face, in-depth, semi-structured interviews. Consolidated Framework for Implementation Research was used to identify facilitators and barriers to implementing physician-pharmacist collaborative clinics for diabetes management in primary healthcare centers, and to explore discriminating factors between low and high implementation units. A sustainable strategy repository based on dynamic sustainability framework was established to inform further implementation.

**Results:**

This study demonstrated that clear recognition of intervention benefits, urgent needs of patients, adaptive and tailored plan, highly collaborative teamwork and leadership support were the major facilitators, while the major barriers included process complexity, large number and poor health literacy of patients in primary areas, inappropriate staffing arrangements, weak financial incentives and inadequate staff competencies. Six constructs were identified to distinguish between high and low implementation units. Sixteen strategies were developed to foster the implementation of physician-pharmacist collaborative clinics, targeting Intervention, Practice setting, and Ecological system.

**Conclusion:**

This qualitative study demonstrated facilitators and barriers to implementing physician-pharmacist collaborative clinics for diabetes management in primary healthcare centers and developed theory-based strategies for further promotion, which has the potential to improve the management of diabetes and other chronic diseases in under-resourced areas.

**Supplementary Information:**

The online version contains supplementary material available at 10.1007/s44197-024-00244-2.

## Introduction

Diabetes has become a major public health problem worldwide, and diabetes-related mortality is increasing rapidly [1]. According to the International Diabetes Federation, the global diabetes prevalence is estimated to be 10.5% in 2021, rising to 12.2% by 2045 [2]. With rapid lifestyle transformation, China has the most people with diabetes about 140 million in 2021, reaching over 174 million by 2045 [2]. Maldistribution of health resources places a heavy burden on diabetes management in healthcare system in China, especially in primary healthcare centers [3]. To achieve maximum utilization and equalization of medical services, the Chinese government has launched a series of strategies to promote the establishment of hierarchical medical system (HMS), which advocates primary healthcare centers to provide diagnosis and treatment of chronic, common and multiple diseases [4, 5]. However, suboptimal utilization and medical quality in primary healthcare centers are the core problems constraining the implementation of HMS [6].

Growing evidence has demonstrated that pharmacists embedded in the physician-pharmacist collaborative care model enhance medication adherence, improve clinical outcomes and decrease medical costs by provide pharmacy services that include patient assessment, medication counseling, comprehensive medication management and patient education for chronic disease [7, 8]. As the first study to introduce physician-pharmacist collaborative clinics for diabetes management into primary healthcare centers in China, we have demonstrated that collaborative clinics could effectively enhance patient medication compliance and quality of life in developing countries [9]. Despite overwhelming evidence, collaborative clinics have not been widely used in under-resourced areas in China [9–11]. Previous studies have shown that scarce support and medical resources hinder service delivery and limit the availability of collaborative clinics to a wider population [9, 12]. Therefore, a deeper understanding of facilitators and barriers, as well as related sustainable implementation strategies, to collaborative clinics implementation may help promote the adoption and reduce medical disparities in under-resourced areas.

Consolidated Framework for Implementation Research (CFIR), integrating extensive implementation models and frameworks, is a popular and practical determinant framework [13]. Containing 5 domains summarizing 39 constructs, CFIR enables a comprehensive and flexible conceptualization and differentiation of facilitators and barriers that influence standardized implementation [14]. Moreover, Expert Recommendations for Implementing Change (ERIC) also reflect to the CFIR constructions, which provides a promising multifaceted strategies repertoire to address potential barriers during targeted implementation [15–17]. A comprehensive study of the determinants of the intervention implementation process and their mapping to recommendations for improvement of the implementation strategy could contribute to the sustainability and generalizability of implementation.

Physician-pharmacist collaborative clinics have been highly recognized around the world, but there are many challenges to their implementation and little evidence to show the sustainability of implementation. This study aims to identify and distinguish the facilitators and barriers of implementing physician-pharmacist collaborative clinics for diabetes management in primary healthcare centers, and further develop strategies for better implementation.

## Methods

### Study Design

This qualitative study was conducted following a multicenter randomized controlled trail in Hunan province in China, with 3 purposively selected primary healthcare center conducting physician-pharmacist collaborative clinics for diabetes management [9]. Located in central China, Hunan province has a wide range of external communication and vast geographical space, which endows sociodemographic and culture diversity. This qualitative study was reported following the Consolidated Criteria for Reporting Qualitative Study (COREQ). This study was approved by the Clinical Research Ethics Committee of the Second Xiangya Hospital of Central South University (No. 2019–213), and all of three primary hospitals accepted the ethic approval. All participants signed informed consent before the qualitative interview.

### Description of the Intervention

Briefly, we established physician-pharmacist collaborative clinics in the intervention group, where physicians provided usual care and pharmacists provided pharmaceutical services. Based on the Theory of Planned Behavior, pharmaceutical intervention program included patient assessment, medication guidance, disease education and patient management, covering the areas of behavior attitude, subjective norm, and perceived behavior control [18]. Patients in the control group received usual care without pharmaceutical intervention. A pragmatic, parallel, multicenter, randomized controlled trial was conducted in May 2021 to evaluate the effectiveness of collaborative clinics in primary healthcare centers in China, and all enrolled patients were asked to attend follow-up visits at 3rd, 6th, 9th, and 12th months [9]. The intervention is detailly described in protocol [19].

### Study Participants and Sampling

Participants in this study of physician-pharmacist collaborative clinics consisted of physicians, pharmacists and patients. Participants were included if they had attended collaborative clinics for 12 months and completed all 4 follow-up visits. Participants were excluded if they refused to audio-record or interview content exceeded 50% off-topic. Purposive sampling techniques with diverse sociodemographic characteristics was used to enroll participants, stratified according to gender, age, educational background, course of the disease, occupation, and years of employment (Supplementary material 1). Recruitment of participants proceeded until data saturation and no additional standpoint emerged, meaning no new codes appeared in the data [20]. All participants were informed about the program and signed consent form, while their privacy and confidentiality were ensured.

### Data Collection

Qualitative data were collected through face-to-face, in-depth, semi-structured interviews, which conducted in separate and calm room without any additional person present. Semi-structured interviews were guided by an interview outline (Supplementary material 2), adapted from the CFIR interview guide [21]. All participants were encouraged to provide their real-life examples derived from their first-hand experience in physician-pharmacist collaborative clinics, rather than prior knowledge. Before the final confirmation, the interview guide was pilot tested and refined in a unit with a total of 7 interviews. The average interview lasted at least 30 min for patients, and 50 min for physicians and pharmacists, and was terminated when no more new information emerged. All audio files were transcribed by 2 researchers respectively.

### Data Analysis

The transcripts were analyzed using analytic inductive and deductive approaches. The researchers reviewed the transcripts and extracted significant statements, summarizing preliminary codes of influencing factors. Then the similar viewpoints were identified and grouped into themes accordingly, conducted by 2 researchers respectively. Conflicting themes and related statements were discussed and agreed upon by a multidisciplinary group. In accordance with the explanation of CFIR guide, themes were deductively mapped into CFIR domains and constructs. Using CFIR rating tool, cross-case comparison of ratings was conducted, reflecting the valence (refers to the influence of the CFIR constructs on the implementation of intervention-positive or negative) and magnitude or strength of each construct, further distinguishing low from high implementation units and identifying the specific barriers to implementation [22]. Constructs were rated by a multidisciplinary group that coded missing as not involved, 0 as no impact or counterbalance of positive and negative impact, + 1/-1 as weak impact or + 2/-2 as strong impact. Criteria used to assign ratings of CFIR constructs was provided (Supplementary material 3). Among all patients in three units participating in physician-pharmacist collaborative clinics, two units had a completion rate of over 70% for patient follow-up visits, while the third unit had a rate of less than 40%. Based on the unit differences in the completion rate, we labeled the 2 units with 70% completion rates as high implementation units and the third as low implementation.

For the strategy construction, we integrated suggestions from participants and mapped them to ERIC strategies using the CFIR-ERIC Strategy Matching Tool [15], followed by adaptation and refinement through multidisciplinary group discussion and consultation with external experts (Supplementary material 4). Furthermore, each barrier and coping strategy were integrated according to Dynamic Sustainability Framework (DSF) and consolidated into a sustainable development implementation framework. Adoption of credibility, transferability, dependability and confirmability criteria to ensure trustworthiness [23]. Maximum differentiation sampling and participant verification was adopted to achieve credibility. Triangulation protocol was conducted to analyze in-depth and multi-perspective interview to ensure transferability. Two researchers transcribed and coded respectively, along with multidisciplinary group discussion and external consultation were performed to ensure dependability and confirmability. All data were analyzed using NVivo 12.

## Results

A total of 43 participants were invited for face-to-face interviews (9 physicians, 12 pharmacists and 22 patients), with response rate of 71.7% (Supplementary material 5). Approximately 90% of physicians have worked for more than 10 years, with half of them working in endocrinology for more than 20 years. Pharmacists working for more than 10 years count about 75%, with 7 working in clinical pharmacy over 5 years (Table 1).

**Table 1:** Demographic Characteristics of Participants

Demographic characteristic	Physicians (*n* = 9)	Pharmacists (*n* = 12)	Patients (*n* = 22)
Gender			
Male	4	3	10
Female	5	9	12
Age, mean (SD), y	43.67(7.62)	37.17(7.37)	55.36(10.78)
Education			
Primary school	0	0	4
Middle school	0	0	10
High school	0	0	4
College	6	7	4
Postgraduate	2	5	0
Doctor	1	0	0
Years of working			
< 10	1	3	NA
10–20	3	5	
20–30	4	3	
30+	1	1	
Years in clinical pharmacy			
< 5	NA	5	NA
5–10		4	
10+		3	

NA: Not applicable

**Fig. 1:**
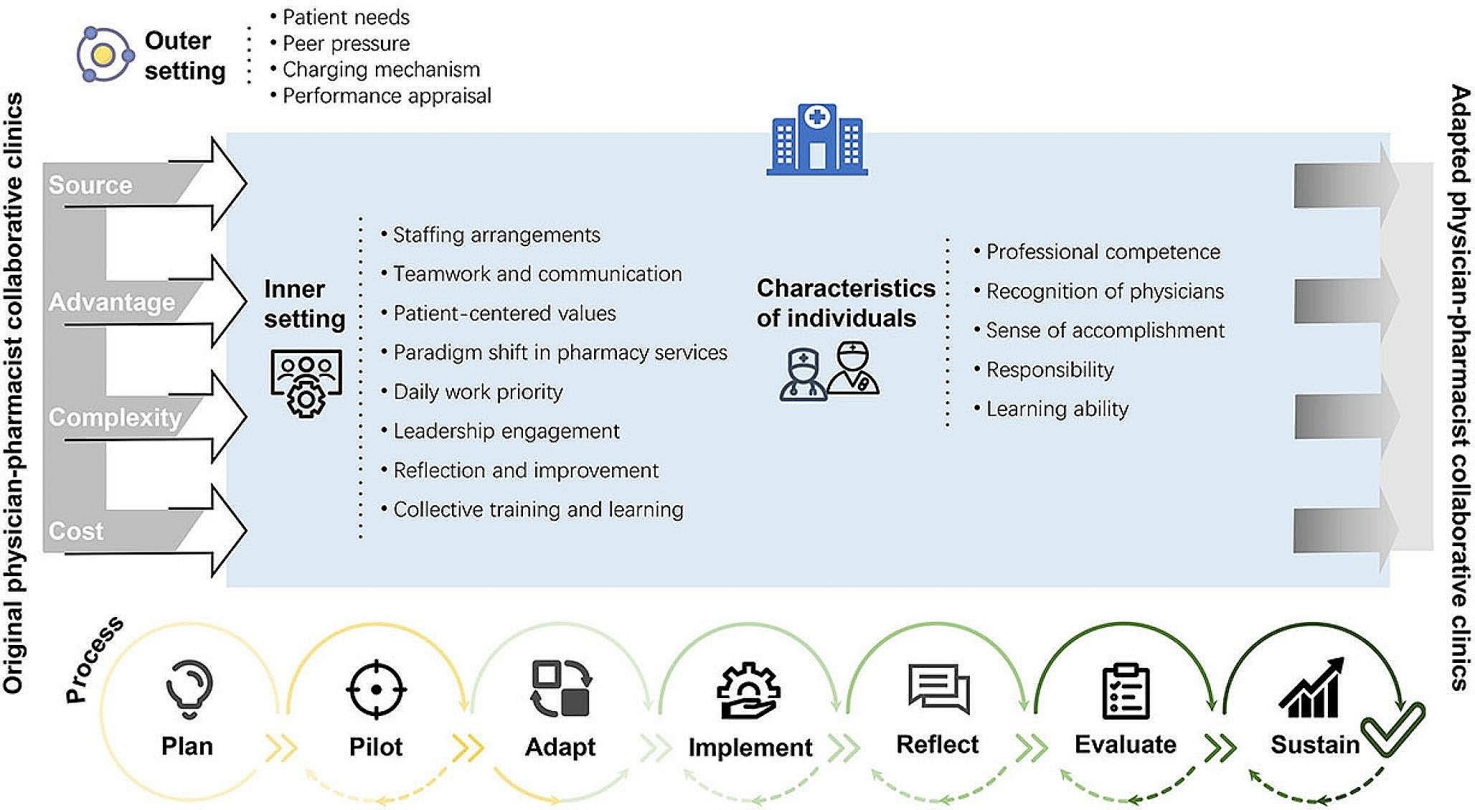
Modified Consolidated Framework for Implementation Research Describing Identified Themes

Similar viewpoints in transcript were summarized into themes, with those having positive emotions for implementation being classified as facilitators and those having negative emotions as barriers. A total of 59 facilitators and 21 barriers of physician-pharmacist collaborative clinics were summarized, mapping to 29 constructs of 5 domains of CFIR framework (Table 2, Supplementary material 6). A modified CFIR framework based on identified themes were constructed in Fig. 1.

**Table 2:** The Facilitators and Barriers Based on CFIR

Domains	Constructs	Facilitators	Barriers
Intervention characteristics	Evidence Strength & Quality	• Evidence of validity	NA
	Relative Advantage	• Timely communication • Complementarity of disciplines	NA
	Adaptability	• Individualized care service	NA
	Trialability	• High recognition of pilot study	NA
	Complexity	NA	• Long-term follow-up • Large number of patients • Complexity of questionnaire
	Design Quality & Packaging	• Patient-centered collaborative care model	NA
	Cost	NA	• Staffing and work time • Dedicated consulting room
Outer setting	Patient Needs & Resources	• Regular follow-up visits and reminders • Pharmacy counseling • Disease education • Medication guidance • Psychological support • Medication cost-effectiveness • mHealth intervention	• Poor health Literacy in primary areas • Distance or transportation difficulties • Unfamiliar with smartphone • Public stigma • Insufficient communication with pharmacists
	Cosmopolitanism	• Enhanced team-based management • online meeting	NA
	Peer Pressure	• Progress report • Informal communication among units	NA
	External Policy & Incentives	• Patient requirements • Incentive policies from study • Leadership support	• Not included in performance appraisal • Lack of charging mechanism
Inner setting	Structural Characteristics	• Clear delineation of workload	• Inappropriate staffing arrangements
	Networks & Communications	• Mature cultivating mode • High quality teamwork • Reporting and discussion	• Lack of communication within the organization
	Culture	• Collective learning • Encouragement of capacity enhancement • Patient-centered values	NA
	Tension for Change	• Paradigm shift in pharmacy services • Irrational drug use in primary • Large patient population and disease burden	NA
	Compatibility	• Responsibility and values	NA
	Relative Priority	• Integration into daily work	• Priority for daily work
	Organizational Incentives & Rewards	• Leadership encouragement • Partner recognition	NA
	Learning Climate	• Positive learning • Acceptance of new things • Reflection and improvement	NA
	Leadership Engagement	• Collective training and learning • Leadership support and oversight • Leaders assist with inter-section communication	• Lack of leadership oversight
	Available Resources	• Dedicated office • Specialist pharmacists	• Inadequate personnel competencies • Conflicting schedules
	Access to Knowledge & Information	• Vocational education • Experience Analysis Sessions	NA
Characteristics of individuals	Knowledge & Beliefs about the Intervention	• Sense of accomplishment and honor • Acceptance of patients	• Unsure convincing
	Self-efficacy	• High professional competence • High recognition of physicians • Better patient outcomes	• Resistance to implement
	Individual Stage of Change	• Career experience accumulation	NA
	Other Personal Attributes	• Internal drive • Competence • Responsibility • Learning ability	NA
Process	Planning	• Preliminary research • Well-established plan • Regular review meeting	NA
	Engaging	• Executive Team • Communication and Discussion	NA
	Executing	NA	• COVID Outbreak

NA: Not applicable

CFIR: Consolidated Framework for Implementation Research

### Intervention Characteristics

Participants reported specific characteristics of physician-pharmacist collaborative clinics of valid evidence, which integrated advantages of various disciplines and could also provide timely communication with patients. “I am very interested in this novel model, because the management of diabetes requires multidisciplinary cooperation” (Physician 2). Thus, collaborative clinics achieved high recognition in pilot phase, which provided patient-center individualized care. “I heard about the collaborative clinics from the literature and online courses. I feel that it was in a good way” (Pharmacist 2). The provision of dedicated personnel and venues provided great convenience for the development of collaborative clinics.

However, general barriers showed that 12-month follow-up appeared to be difficult for participants to adhere to, indicating that patients in primary areas had not developed the concept of regular follow-up visit for chronic diseases. “It’s difficult for patients in rural areas to understand the content of education, and some elderly people even have communication difficulties” (Pharmacist 10). Other barriers to implementing collaborative clinics were staffing arrangement and dedicated room, exacerbated by large patient populations. “The number of patients is snowballing. Sometimes we can’t keep up” (Physician 4).

### Outer Setting

Patients reported that collaborative care mode met their needs for regular follow-up, pharmacy counseling, mediation guidance and education, which served as important external facilitators to incentivize the implementation of collaborative clinics. “When I have poor blood sugar control, I see my pharmacist to adjust the dose of medication” (Patient 12). However, patients in primary areas are characterized by low health literacy, large proportion of elderly people, unfamiliarity with smart devices, and inconvenient transportation, which greatly hinders the promotion among primary patients. “Some patients have poor literacy of health, which may be due to a lower level of education in the villages” (Pharmacist 5).

Despite a solid collaborative team, regular meetings and reports, and leadership support drive the implementation, participants acknowledged that the absence of pharmacy-based charging and performance appraisal are major barriers. “Pharmacy charge is a big deal” (Pharmacist 3). “I’m very busy with my regular job and only do patient management when I have free time” (Physician 2).

### Inner Setting

Evidence showed that units with clear delineation of work, collaborative network, regular communication, and shared values were better at implementation. “I have organized each pharmacist’s role in such a way that there is good cooperation” (Pharmacist 3). Despite irrational drug use and poor medication compliance in primary areas prompted a paradigm shift in pharmacy services, collaborative clinics were not integrated into daily work practice and lacked leadership oversight, resulting in low implementation in several units. “I’m embarrassed that I haven’t done much work as a leader” (Pharmacist 8).

Encouragement from leaders and recognition from partners have led pharmacists to actively engage in self-learning, improve work patterns, and participate in professional training, with the support and supervision of leaders playing a very important role. “I use online courses to learn my expertise, and my leaders give us platforms and opportunities to learn as well” (Pharmacist 2). However, inappropriate staffing arrangements, inadequate competency and less communication impeded implementation in several units. “Pharmacists used their spare time to follow up with patients, but they are already multi-tasking” (Pharmacist 9).

### Characteristics of Individuals

Specific characteristics of health professionals tremendously influenced the implementation. These factors, including sense of accomplishment and honor for improving patient outcomes, desire for personal empowerment, and high physician recognition, contribute greatly to the enthusiasm of pharmacists to implement. “I’m just in the way of skill accumulation and learning. The more I learn, the better I could help clinical physicians” (Pharmacist 9).

Participants at all centers acknowledged that resistance to implementation was the main barrier due to increased workload and difficulty in communicating with patients. Inexperienced pharmacists showed low conviction in implementing. “I don’t think I’m doing a perfect job because I also have a lot of daily work, so sometimes I don’t follow up with patients timely” (Pharmacist 6).

### Process

Various measures were adopted to ensure the implementation, including preliminary adaptation study, implementation plan and regular review meetings. Moreover, implementation units established executive teams, consisting of leadership, executive leader, specialist physicians and pharmacists, and organized team debriefings and discussions. “We will be criticized for our failure to accomplish the task at the meeting.” (Pharmacist 8).

However, the outbreak of COVID severely disrupted the implementation plan, resulting in fewer visits by the diabetic population due to fear of infection. “It seriously affects the trial process. Because some patients had found the process of care-seeking complicated, it’s even more so during the epidemic” (Pharmacist 10).

### CFIR Constructs Rating

Construct rated based on the viewpoints emotions under the summarized themes, and we provided more detailed descriptions of discriminative constructs between high and low implementation (Supplementary material 7). Of the 29 CFIR constructs assessed, 5 constructs strongly discriminated between high and low implementation units, while another 1 construct exhibited a weak discriminant (Table 3). The construct of *patient needs and resources* in the outer setting discriminated high and low implementation units weakly. The majority of strong discriminative constructs were related to the inter setting, including *structural characteristics*, *networks and communications*, *relative priority*, *learning climate* and *leadership engagement*. Obviously, high implementation units characterized by solid staffing architecture, multifaceted internal collaboration, urgency for implementation, positive learning atmosphere, and leadership support.

**Table 3:** Ratings Assigned to CFIR Construct by Case

Domains	Constructs	High implementation units		Low implementation units	Distinguishing constructs
		Unit 1	Unit 2	Unit 3	
Intervention characteristics	Evidence Strength & Quality	+ 2	+ 1	+ 1	Not
	Relative Advantage	+ 2	+ 2	+ 1	Not
	Adaptability	+ 2	+ 2	+ 1	Not
	Trialability	+ 1	Missing	Missing	Not
	Complexity	-1	-2	-2	Not
	Design Quality & Packaging	Missing	Missing	+ 1	Not
	Cost	-1	-1	-2	Not
Outer setting	Patient Needs & Resources	+ 1	+ 1	0	Weak
	Cosmopolitanism	+ 1	+ 2	+ 1	Not
	Peer Pressure	+ 1	0	+ 2	Not
	External Policy & Incentives	+ 2	+ 1	+ 1	Not
Inner setting	Structural Characteristics	+ 1	+ 1	-2	Strong
	Networks & Communications	+ 1	+ 2	-2	Strong
	Culture	+ 2	+ 1	+ 1	Not
	Tension for Change	+ 2	+ 1	+ 2	Not
	Compatibility	+ 1	0	+ 1	Not
	Relative Priority	+ 2	+ 1	-1	Strong
	Organizational Incentives & Rewards	+ 2	0	+ 1	Not
	Learning Climate	+ 2	+ 1	0	Strong
	Leadership Engagement	+ 2	+ 1	-1	Strong
	Available Resources	+ 1	-1	-1	Not
	Access to Knowledge & Information	+ 1	0	+ 1	Not
Characteristics of individuals	Knowledge & Beliefs about the Intervention	+ 2	+ 1	+ 1	Not
	Self-efficacy	+ 2	+ 1	+ 1	Not
	Individual Stage of Change	+ 1	0	+ 1	Not
	Other Personal Attributes	+ 1	+ 1	+ 1	Not
Process	Planning	+ 1	+ 1	0	Not
	Engaging	+ 2	+ 2	+ 1	Not
	Executing	-1	-1	-2	Not

CFIR: Consolidated Framework for Implementation Research

### Strategy Design

**Fig. 2:**
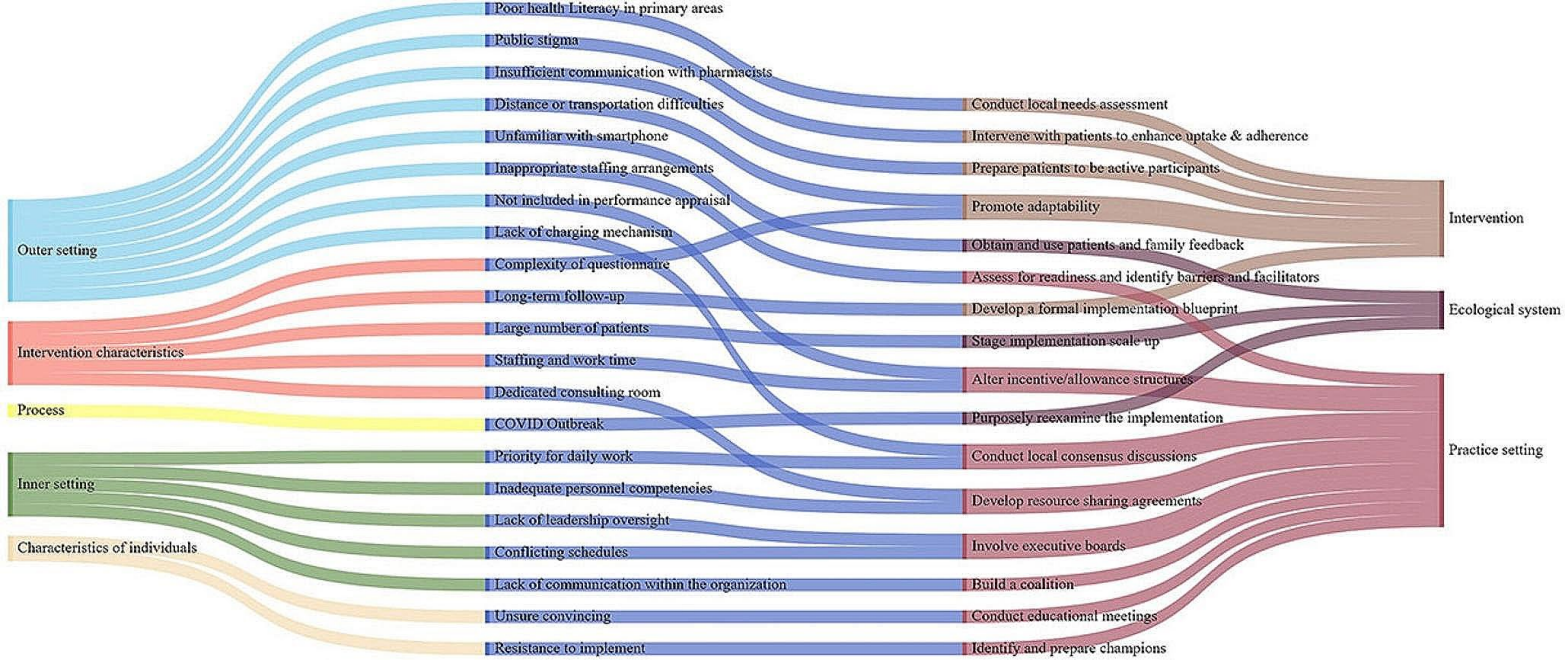
Identified Barriers and Strategies in 3 Components of the Dynamic Sustainability Framework

Based on the 21 identified barriers to the implementation of collaborative clinics, we mapped them to the CFIR-ERIC Strategy Matching Tool. Sixteen strategies were discussed by muti-disciplinary panel, which were systematically integrated by the DSF framework for sustainable implementation, consisting of 3 domains of Intervention, Practice setting, and Ecological system (Fig. 2).

In particular, adoption of the intervention reflects that positive adaptation for sustainable implementation requires early integration of local needs to ensure patient acceptance and activation, highlighting assessment local needs, improving patient adherence, stimulating patient initiative, promoting adaptability and conducting local formal blueprint for intervention implementation. Targeting the internal climate of the implementation unit, Practice setting aims to adapt its characteristics to the delivery of the intervention, while strategies include assessing readiness, altering incentive structures, establishing local consensus, sharing resources, involving executive board and champions, building coalitions, and conducting educational meetings. During the implementation process, proper maintenance of Ecological system is a critical of factor for sustainability. Thus, scaling up implementation, obtaining timely feedback from participants and evaluating implementation are at the core of sustainable implementation of the intervention system.

## Discussion

This qualitative study was conducted by multidisciplinary group to multifacetedly reflect the facilitators and barriers in physician-pharmacist collaborative clinics using CFIR tool, further explore the discriminative constructs between low and high implementation and establish sustainable implementation strategy based on DSF construction. Influencing factors covered 5 domains and 29 constructs of CFIR, in which clear recognition of intervention benefits, urgent patient needs, adaptive and tailored plan, highly collaborative teamwork and leadership support were the major facilitators, while the major barriers included process complexity, large number and poor health literacy of patients in primary areas, inappropriate staffing arrangements, weak financial incentives and inadequate personnel competencies. This study identified 6 CFIR constructs to distinguish high and low implementation units, and developed 16 strategies to overcome these challenges.

Main barriers in implementing intervention focused on available resources, local patient needs and resources, staff arrangement and communications, and executing process. Implementation units have also taken numerous measures to address these challenges, including developing individualized care services to promote patient adaptability, adding mHealth interventions to enhance patient engagement, requesting dedicated offices and training staff. Generally, these measures are applicable to other primary healthcare centers implementing new interventions. Moreover, a notification on the fee-charging criteria for pharmacy services was issued in October 2023, which will tremendously motivate and inspire the practice of pharmacists [24]. It is foreseeable that improvement of pharmacy services could ultimately benefit patients and optimize the utilization of medical resource.

In this study, we identified 5 strong and 1 weak CFIR constructs to distinguish between high and low implementation units. Weak discriminant construct nested in the outer setting, with strong discriminant constructs in the inner setting. Our findings were supported by an implementation study of a weight management program, which showed that the majority of discriminative constructs were associated with inner setting [22]. Evidence recognized the core influential roles of leadership engagement, which dominated in provision of dedicated office and staffing arrangements to satisfy patient needs and develop strong communication within units, resulting in a high relative priority of intervention [22, 25]. Our findings of leadership engagement, communication and work priority were similar to studies in developed countries, but we firstly explored that low implementation units in primary healthcare centers in China are characterized by unstable organizational structures and insufficient learning atmosphere. A national cross-sectional study in 2017 demonstrated that the coverage of clinical pharmacy services was unsatisfactory in 81.75% of primary healthcare centers and staff composition was unqualified in 57.73% [26]. Underestimating of professional training demand could hurt the learning climate of organization and organizational commitment, leading to low job performance and prevalence of exhaustion [27, 28]. Therefore, professional training for pharmacists and improving of pharmacy service system could be the core measurements in the future.

In order to improve the efficiency of physician-pharmacist collaborative clinics, we developed local implementation strategy repository based on DSF, aiming to institutionalize interventions within local organizations. Undoubtedly, with substantial resources devotion and successful intervention implementing, building necessary capacity to support sustainable delivery has been meaningful [29]. The DSF highlights that variation occurred in application of interventions over time, in the characteristics of practice settings and in broader systems that provides environment for care delivery, and has been used in identifying threats to sustainability, predicting sustainable implementing, and evaluating adaptation of interventions [30, 31].

DSF highlights that dynamic changes influence the ability of health interventions which exists in the evidence-based practice [32]. In this study, patients in primary areas characterized by misunderstanding of diabetes and poor health literacy, which may lead to low rates of awareness, treatment and glucose control of diabetes and unsatisfactory outcomes of diabetes management in China [33, 34]. Studies showed that lack of environmental resources and strategies were main barriers to diabetes self-management [35]. An analysis of big data in primary areas in China showed that existing diabetes management models failed to customize management strategies from the patient’s perspective and ignore patient needs [36]. Consistent with our previous research, studies have shown that physician-pharmacist collaborative clinics for diabetes management significantly improve patient medication compliance and clinical outcomes in primary areas [9, 37, 38]. Despite the effectiveness of collaborative clinics is widely recognized and proven, for further implementation in primary areas, it’s necessary to incorporate patient education appropriate to the cultural context and native language, utilize telephone calls and home visits to enhance the participation of patients’ families, and develop local programs for diabetes control in primary areas, which may be effective in increasing patient acceptance and participation [39, 40].

The practice setting ultimately determines the sustainable extent of intervention implementation and ultimate benefit, typically including context characteristics, culture and process [30]. Studies demonstrated that role ambiguity and conflict were common among pharmacists in China, which means lack of responsibility definition, performance evaluation criteria and conflict between different expectations, resulting in a negative efficiency and quality of pharmacy services [41]. The improvement of healthcare policies and resource support, together with development of pharmacist team would be so difficult due to excessive dedication, but professional training represents a less resource-intensive approach and is relatively easy to implement, in which skills of pharmaceutical care service were the most mentioned [42, 43]. Moreover, lack of reimbursement for pharmacy services hindered the enthusiasm of pharmacists, resulting in unattractive wages and career development [26, 44]. As a positive measure, pay-for-performance for professionalism patient-centered care in diabetes management could be utilized [45]. It is clear that administrative and executive support was crucial in implementation of interventions, which was reflected in the dedication of resources and incentives [46]. Therefore, promoting and demonstrating the importance of pharmacy services in primary areas would facilitate the transition and development of pharmacy.

DSF recognized ecological system as extra driver of successfully implementation and sustainability of interventions, interacting with practice settings and interventions, which includes population characteristics, outer setting and market forces [30]. Actually, primary healthcare centers serve over 900 million people in China, with 12.0% diabetic population, resulting in professional staff shortages and heavy workloads [33, 47]. Multidisciplinary care combined with mobile technology was promising to solve relative understaffing and to increase the connection between health professionals and patients and their families [48]. Moreover, implementation scale-up and timely feedback from participants could help ensure and improve the quality of the intervention. Online evaluation application and regular satisfaction survey would be essential part for sustainability [49]. Despite multiple contingencies considered at the beginning of the trial design, the COVID epidemic disrupted our plan, suggesting that a reexamination system should be established.

### Strengths and Limitations

First, strengths of our study included our primary care-level design and advanced approach, involving patients participating 12-month follow-up visits in physician-pharmacist collaborative clinics in three counties, and guided by robust implementation framework. This work reflects the dilemma of hierarchical medical system in primary healthcare and shows the way forward for chronic disease management in under-resourced areas. Second, our large sample size of 43 multi-perspective interviews corroborated the whole process of implementing novel management mode, and the utilizing of implementation rating tool illustrated internal differences of implementation units, contributing to multidisciplinary strategy repository. Future study may focus on implementation differences to construct targeted sustainability strategies. Third, this study systematically innovated the research method based on CFIR framework. We constructed a modified CFIR framework and shed light on the application of the CFIR. However, the findings focused on the influencing factors of physician-pharmacist collaborative clinics for diabetes management, potentially limiting its generalization to other populations. The COVID epidemic affected patients’ willingness to visit clinics, and this effect should be further investigated.

Physician-pharmacist collaborative clinics for diabetes have been shown to significantly improve patient management, delay disease complications and reduce medical burden [7, 8]. Due to limited resources and poor implementation in primary healthcare centers in China, the identification of associated facilitators and barriers, as well as the development of implementation strategies, were needed to ensure the sustainability of effectiveness of collaborative clinics. To our knowledge, this is the first study to explore the sustainability of physician-pharmacist collaborative clinics, particularly in the context of the trend towards multidisciplinary collaboration. It is undeniable that most countries in the Western Pacific region are still in the underdevelopment stage and have a shortage of medical resources, while changes in people’s lifestyles are increasing the incidence of chronic diseases such as diabetes, further straining medical resources. Therefore, multidisciplinary team collaboration in chronic disease management is of great importance in areas with scarce medical resources.

## Conclusion

This study demonstrated facilitators and barriers to implementing physician-pharmacist collaborative clinics for diabetes management in primary healthcare centers in China, and identified six discriminant constructs of CFIR, through a synthesis of semi-structured interviews and discussions. Sixteen strategies for sustainable implementation were developed as a theory-based response to promote the implementation of physician-pharmacist collaborative clinics in primary healthcare settings. This study is promising for improving the implementation of physician-pharmacist collaborative care model for diabetes and other chronic diseases in under-resourced areas.

## Electronic Supplementary Material

Below is the link to the electronic supplementary material.

Supplementary Material 1

## Data Availability

The qualitative data are not publicly available because the information may compromise the privacy of research interviewees. Data are available from the corresponding author on reasonable request.
