# Supplementary material for: Sustainable Implementation of Physician-Pharmacist Collaborative Clinics for Diabetes Management in Primary Healthcare Centers: A Qualitative Study
Source: J Epidemiol Glob Health. 2024 May 23;14(3):974–86. doi: 10.1007/s44197-024-00244-2 (PMC11442712; doi:10.1007/s44197-024-00244-2)
Supplement: Supplementary file 1 — Supplementary Material 1 [file 44197_2024_244_MOESM1_ESM.pdf]

Supplementary materials

Supplementary material 1 Purposive sampling techniques

Supplementary material 2 Interview guide

Supplementary material 3 Criteria for constructs rating

Supplementary material 4 Results of matching tool

Supplementary material 5 Study and participant flowchart of the study

Supplementary material 6 The facilitators and barriers of implementing physician-pharmacist collaborative clinics for diabetes management

Supplementary material 7 The ratings of discriminative constructs of CFIR between high and low implementation units

Supplementary material 1 Purposive sampling techniques

Characteristics	Patients	Physicians and pharmacists
Gender ratio	1.00-1.25	1.00-1.25
Age, y	18-75	NA
Education	Primary, Meddle, High school, College or above	College, Postgraduate, Doctor
Course of disease, y	<5, 5-10, 11-15, >15	NA
Occupation	Non-manual, Few-manual, Manual worker	NA
Years of work	NA	<10, 10-20, 20-30, >30
Years in clinical pharmacy	NA	<5, 5-10, >10

NA: Not applicable.

Supplementary material 2 Interview guide

1 Interview guide in patient

1.1 Patient experiences of participating in Physician-Pharmacist collaborative clinics

Q1: Have you ever been to the Physician-Pharmacist collaborative clinics before our clinical trial?

What kind of Physician-Pharmacist collaborative clinics did you attend?

Q2: Why did you participate in Physician-Pharmacist collaborative clinics?

What do you expect from the Physician-Pharmacist collaborative clinics?

Has your expectation been fulfilled?

Q3: How do you feel about the Physician-Pharmacist collaborative clinics?

What is the difference between the Physician-Pharmacist collaborative clinics and the usual clinic you visited before?

Do you think visits in Physician-Pharmacist collaborative clinic are helpful for your diabetes treatment? Can you give me an example?

Q4: What services does the pharmacist provide in the Physician-Pharmacist collaborative clinics? Can you describe in detail?

What do you think of the services?

Which services are great? What other services do you need?

Q5: How would you rate pharmacists' work in Physician-Pharmacist collaborative clinics?

Are pharmacists up to the job?

Q6: Do you feel comfortable communicating with pharmacists?

Are you willing to accept the pharmacist's advices and strictly implement them?

Which advice have not been implemented? Can you tell me the reasons?

Q7: What is the impact of Physician-Pharmacist collaborative clinics on patients compared to usual clinics?

What conveniences and inconveniences does the Physician-Pharmacist collaborative clinics bring to you? Can you tell me the reasons?

1.2 Patient willingness to promote the implementation of Physician-Pharmacist collaborative clinics

Q8: After the follow-ups of our project, if the hospital conducts a Physician-Pharmacist collaborative clinic, are you willing to choose?

Are you willing to introduce other diabetic patients to the Physician-Pharmacist collaborative clinics?

Q9: What other diseases can be treated by Physician-Pharmacist collaborative clinics?

Q10: What advice do you have for Physician-Pharmacist collaborative clinics?

2 Interview guide in physician

2.1 Physician experiences of participating in Physician-Pharmacist collaborative clinics

Q1: Will you introduce treatment schedule to the patient when prescribing in usual clinics?

How are your patients performing in your treatment schedules? /Are they executing your treatment schedules properly?

How are the patients' compliance?

What is the difference between patients participating in Physician-Pharmacist collaborative clinics and usual clinics?

Q2: How would you rate pharmacists' work in Physician-Pharmacist collaborative clinics?

When will you communicate with pharmacists/ask the pharmacists' opinion?

Would you take advices of pharmacists? Can you give me an example?

Q3: Do you feel comfortable communicating with pharmacists?

What problems did you encounter in your communication with the pharmacists?

Q4: What do you think of the pharmaceutical services provided by the pharmacists in Physician-Pharmacist collaborative clinics?

What does pharmacists do in Physician-Pharmacist collaborative clinics?

What are the advantages and disadvantages of Physician-Pharmacist collaborative clinics?

What kind of work do pharmacists need to do? /What advice do you have for pharmacists' work?

If pharmacists want to provide pharmaceutical services, which way is the most direct and effective?

How has your perception of pharmacists changed through Physician-Pharmacist collaborative clinics?

Q5: What is the impact of Physician-Pharmacist collaborative clinics on physicians compared to usual clinics?

What conveniences and inconveniences does the Physician-Pharmacist collaborative clinics bring to you? Can you tell me the reasons?

Q6: What is the impact of Physician-Pharmacist collaborative clinics on patients compared to usual clinics?

2.2 Physician willingness to promote the implementation of Physician-Pharmacist collaborative clinics

Q7: After the follow-ups of our project, if the hospital conducts a Physician-Pharmacist collaborative clinic, are you willing to participate in?

Q8: What other diseases can be treated by Physician-Pharmacist collaborative clinics?

Q9: What advice do you have for Physician-Pharmacist collaborative clinics?

Q10: What are the enablers and barriers in promoting the implementation of Physician-Pharmacist collaborative clinics in primary hospitals?

3 Interview guide in pharmacist

3.1 Pharmacist experiences of participating in Physician-Pharmacist collaborative clinics

Q1: What are your main responsibilities as a clinical pharmacist/pharmacist?

Have you ever known Physician-Pharmacist collaborative clinics? / What are the differences between what you learned and what you experienced?

What kind of work do you mainly undertake in Physician-Pharmacist collaborative clinics?

Q2: Do you think the communication with physicians is smooth and comfortable?

What problems did you encounter in your communication with the physicians?

When did you communicate with physicians?

Would you give advises to physicians? Did they accept? Can you give an example?

Q3: Do you feel comfortable communicating with patients?

Did the patients accept your advice and implement it accurately? Can you give an example?

How were the patients' compliance?

Q4: How has the Physician-Pharmacist collaborative clinics affected pharmacists?

How would you rate your work?

Q5: What is the impact of Physician-Pharmacist collaborative clinics on pharmacists compared to usual clinics?

How will you improve your work?

Do you have other ways to provide pharmaceutical services?

3.2 Pharmacist willingness to promote the implementation of Physician-Pharmacist collaborative clinics

Q6: Did any patient come to you for consultation in outpatient clinic?

What did patients ask you about?

Q7: After the follow-ups of our project, if the hospital conducts a Physician-Pharmacist collaborative clinic, are you willing to participate in?

Q8: What other diseases can be treated by Physician-Pharmacist collaborative clinics?
Q9: What advice do you have for Physician-Pharmacist collaborative clinics?
Q10: What are the enablers and barriers in promoting the implementation of Physician-Pharmacist collaborative clinics in primary hospitals?
Q11: What would pharmacists do to promote the implementation of Physician-Pharmacist collaborative clinics?

Supplementary material 3 Criteria for constructs rating

Rating	Criteria
−2	The construct is a negative influence in the organization, an impeding influence in work processes, and/or an impeding influence in implementation efforts. The majority of interviewees (at least two) describe explicit examples of how the key or all aspects (or the absence) of a construct manifests itself in a negative way.
−1	The construct is a negative influence in the organization, an impeding influence in work processes, and/or an impeding influence in implementation efforts. Interviewees make general statements about the construct manifesting in a negative way but without concrete examples
	• The construct is mentioned only in passing or at a high level without examples or evidence of actual, concrete descriptions of how that construct manifests;
	• There is a mixed effect of different aspects of the construct but with a general overall negative effect;
	• There is sufficient information to make an indirect inference about the generally negative influence;
	• Judged as weakly negative by the absence of the construct.
0	A construct has neutral influence if:
	• It appears to have neutral effect (purely descriptive) or is only mentioned generically without valence;
	• There is no evidence of positive or negative influence;
	• Credible or reliable interviewees contradict each other;
	• There are positive and negative influences at different levels in the organization that balance each other out; and/or different aspects of the construct have positive influence while others have negative influence and overall, the effect is neutral.
+1	The construct is a positive influence in the organization, a facilitating influence in work processes, and/or a facilitating influence in implementation efforts. Interviewees make general statements about the construct manifesting in a positive way but without concrete examples:
	• The construct is mentioned only in passing or at a high level without examples or evidence of actual, concrete descriptions of how that construct manifests;
	• There is a mixed effect of different aspects of the construct but with a general overall positive effect;
	• There is sufficient information to make an indirect inference about the generally positive influence.
+2	The construct is a positive influence in the organization, a facilitating influence in work processes, and/or a facilitating influence in implementation efforts. The majority of interviewees (at least two) describe explicit examples of how the key or all aspects of a construct manifests itself in a positive way.
	Missing Interviewee(s) were not asked about the presence or influence of the construct; or if asked about a construct, their responses did not correspond to the intended construct and were instead coded to another construct. Interviewee(s) lack of knowledge about a construct does not necessarily indicate missing data and may instead indicate the absence of the construct.

Supplementary material 4 Results of matching tool

ERIC Strategies	Complexity	Cost	Patient Needs & Resources	External Policy & Incentives	Structural Characteristics	Networks & Communications	Relative Priority	Leadership Engagement	Available Resources	Knowledge & Beliefs about the Intervention	Self-efficacy	Executing
Respondents (Denominator)	30	25	21	27	22	23	28	22	23	25	27	29
Alter incentive/allowance structures	7%	44%	10%	41%	18%	0%	39%	32%	17%	16%	4%	17%
Alter patient/consumer fees	0%	20%	0%	7%	0%	0%	0%	0%	22%	0%	0%	0%
Assess for readiness and identify barriers and facilitators	30%	16%	33%	4%	36%	13%	36%	14%	13%	20%	11%	31%
Audit and provide feedback	3%	8%	5%	0%	5%	17%	14%	5%	0%	4%	22%	17%
Build a coalition	0%	4%	14%	33%	27%	39%	18%	18%	17%	16%	0%	0%
Capture and share local knowledge	27%	4%	10%	26%	23%	26%	14%	9%	22%	24%	19%	14%
Centralize technical assistance	10%	0%	0%	0%	5%	26%	0%	5%	0%	0%	11%	7%
Change accreditation or membership reqs	0%	4%	0%	15%	5%	0%	4%	5%	0%	0%	0%	0%
Change liability laws	0%	0%	0%	19%	0%	0%	0%	0%	0%	0%	0%	0%
Change physical structure and equipment	3%	4%	0%	0%	32%	0%	0%	0%	48%	0%	0%	3%
Change record system	0%	0%	5%	4%	9%	9%	7%	0%	4%	0%	4%	7%
Change service sites	0%	0%	0%	4%	14%	0%	0%	0%	4%	0%	0%	0%
Conduct cyclical small tests of change	37%	8%	10%	4%	23%	9%	4%	0%	13%	12%	26%	7%
Conduct educational meetings	13%	12%	10%	15%	5%	13%	7%	9%	0%	56%	15%	3%
Conduct educational outreach visits	7%	4%	5%	0%	0%	4%	14%	9%	0%	28%	22%	14%
Conduct local consensus discussions	7%	4%	29%	22%	14%	22%	46%	27%	0%	12%	0%	7%
Conduct local needs assessment	3%	4%	57%	7%	18%	9%	32%	14%	0%	24%	0%	3%
Conduct ongoing training	37%	0%	0%	4%	0%	4%	4%	0%	9%	12%	41%	28%
Create a learning collaborative	33%	8%	0%	15%	18%	35%	4%	5%	9%	16%	30%	21%
Create new clinical teams	3%	0%	10%	0%	9%	13%	0%	0%	4%	0%	7%	7%
Create or change credentialing and/or licensure standards	0%	4%	0%	19%	5%	0%	14%	0%	4%	0%	0%	3%
Develop a formal implementation blueprint	43%	8%	5%	7%	18%	13%	14%	23%	4%	4%	11%	28%
Develop academic partnerships	0%	4%	5%	11%	5%	9%	0%	0%	4%	12%	7%	0%
Develop an implementation glossary	3%	4%	0%	4%	9%	0%	0%	5%	0%	4%	0%	3%
Develop and implement tools for quality monitoring	7%	0%	14%	11%	5%	0%	7%	9%	0%	0%	4%	31%
Develop and organize quality monitoring systems	10%	4%	0%	15%	5%	0%	0%	5%	0%	0%	7%	21%
Develop disincentives	0%	16%	0%	7%	5%	0%	7%	23%	0%	0%	0%	0%
Develop educational materials	13%	0%	10%	4%	0%	0%	7%	0%	4%	36%	19%	7%
Develop resource sharing agreements	0%	32%	0%	0%	5%	4%	0%	0%	26%	0%	4%	0%
Distribute educational materials	3%	0%	5%	0%	0%	4%	4%	0%	0%	16%	4%	7%
Facilitate relay of clinical data to providers	3%	0%	10%	4%	0%	4%	7%	5%	0%	12%	7%	10%
Facilitation	20%	8%	0%	4%	9%	26%	14%	18%	4%	20%	22%	24%
Fund and contract for clinical innovation	3%	28%	0%	15%	14%	0%	11%	18%	39%	4%	4%	3%
Identify and prepare champions	30%	12%	5%	22%	27%	17%	18%	41%	4%	40%	30%	14%
Identify early adopters	20%	8%	0%	7%	23%	17%	7%	9%	0%	20%	19%	14%
Increase demand	3%	12%	10%	0%	0%	0%	29%	27%	4%	20%	0%	0%
Inform local opinion leaders	13%	12%	0%	22%	14%	22%	14%	18%	0%	28%	4%	3%
Intervene with patients/consumers to enhance uptake & adherence	3%	4%	24%	4%	0%	0%	4%	14%	0%	0%	0%	3%
Involve executive boards	0%	20%	5%	41%	14%	9%	11%	45%	17%	0%	0%	7%
Involve patients/consumers and family members	0%	0%	71%	11%	9%	9%	18%	9%	0%	0%	4%	3%
Make billing easier	3%	32%	0%	7%	0%	0%	4%	5%	22%	0%	0%	3%
Make training dynamic	10%	0%	0%	0%	5%	0%	7%	9%	0%	0%	41%	3%
Mandate change	7%	8%	0%	15%	5%	4%	32%	14%	0%	4%	4%	0%
Model and simulate change	27%	20%	0%	4%	14%	4%	0%	14%	0%	4%	33%	14%
Obtain and use patients/consumers and family feedback	0%	4%	76%	0%	5%	0%	7%	14%	0%	4%	4%	0%
Obtain formal commitments	0%	0%	0%	15%	9%	9%	14%	27%	13%	0%	0%	14%
Organize clinician implementation team meetings	20%	0%	0%	0%	14%	52%	4%	0%	9%	4%	11%	24%
Place innovation on fee for service lists/formularies	0%	24%	0%	19%	0%	0%	7%	14%	17%	0%	0%	0%
Prepare patients/consumers to be active participants	0%	0%	48%	0%	0%	0%	0%	5%	0%	0%	0%	0%
Promote adaptability	40%	16%	14%	0%	23%	0%	18%	9%	4%	16%	11%	10%
Promote network weaving	0%	0%	0%	11%	23%	57%	4%	9%	9%	12%	4%	0%
Provide clinical supervision	7%	0%	5%	0%	0%	4%	0%	0%	0%	0%	11%	3%
Provide local technical assistance	17%	4%	5%	7%	18%	9%	0%	5%	0%	0%	22%	31%
Provide ongoing consultation	20%	0%	5%	0%	9%	0%	14%	14%	0%	4%	41%	24%
Purposely reexamine the implementation	17%	0%	5%	11%	0%	4%	4%	9%	4%	4%	0%	45%
Recruit, designate and train for leadership	7%	4%	0%	0%	18%	17%	11%	23%	4%	4%	4%	7%
Remind clinicians	0%	0%	0%	0%	0%	4%	4%	5%	0%	0%	0%	3%
Revise professional roles	3%	0%	0%	4%	18%	0%	0%	5%	9%	0%	0%	7%
Shadow other experts	7%	0%	0%	0%	5%	0%	0%	0%	0%	4%	33%	0%
Stage implementation scale up	30%	8%	0%	4%	14%	0%	7%	0%	13%	20%	15%	7%
Start a dissemination organization	0%	0%	5%	0%	9%	13%	0%	0%	0%	4%	0%	0%
Tailor strategies	27%	12%	14%	11%	18%	4%	14%	5%	9%	12%	11%	10%
Use advisory boards and workgroups	0%	0%	29%	15%	5%	13%	7%	18%	4%	8%	7%	10%
Use an implementation adviser	10%	4%	5%	4%	5%	9%	7%	18%	13%	0%	7%	21%
Use capitated payments	0%	16%	0%	0%	0%	0%	4%	5%	9%	0%	0%	0%
Use data experts	3%	12%	0%	4%	0%	4%	7%	0%	0%	4%	4%	0%
Use data warehousing techniques	0%	0%	5%	0%	0%	0%	0%	5%	0%	0%	0%	0%
Use mass media	0%	4%	0%	15%	0%	9%	7%	0%	0%	8%	0%	0%
Use other payment schemes	0%	20%	0%	15%	0%	0%	7%	5%	22%	0%	0%	0%
Use train the trainer strategies	7%	0%	0%	4%	0%	9%	0%	0%	9%	4%	15%	3%
Visit other sites	3%	16%	0%	7%	5%	4%	4%	5%	9%	12%	15%	17%
Work with educational institutions	0%	4%	0%	4%	5%	4%	0%	0%	4%	4%	0%	0%
Access new funding	3%	72%	0%	7%	5%	4%	11%	9%	78%	8%	0%	3%

Supplementary material 5 Study and participant flowchart of the study

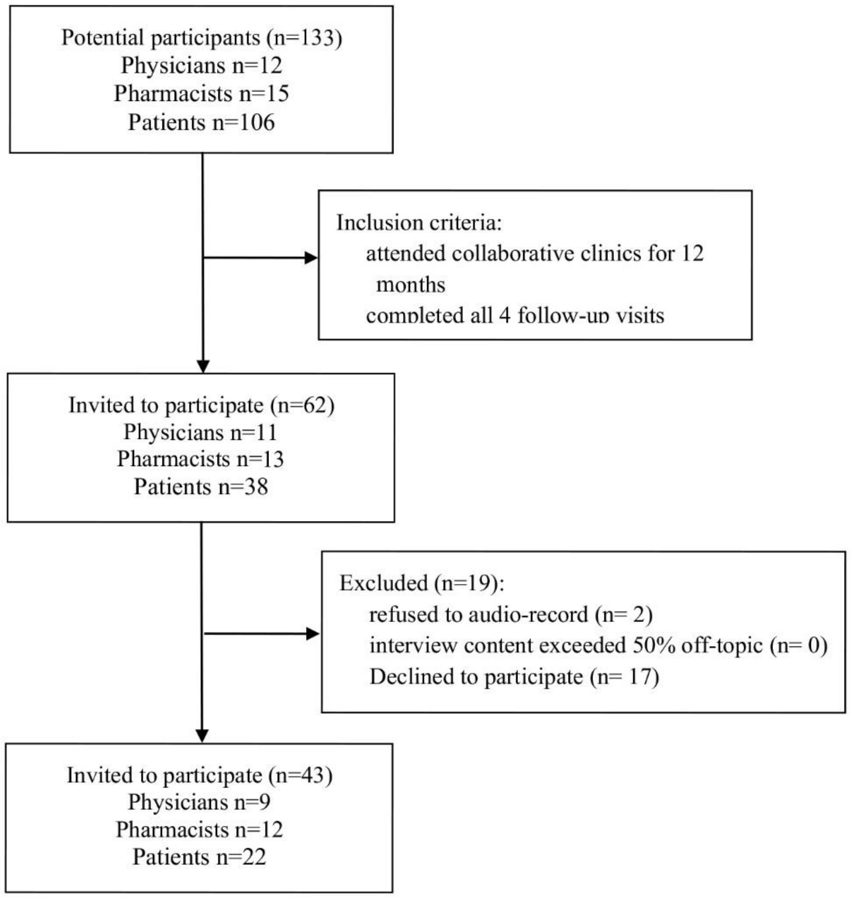

Supplementary material 6 The facilitators and barriers of implementing physician-pharmacist collaborative clinics for diabetes management

Domain	Construct	Facilitators	Barriers
Intervention characteristics	Evidence Strength & Quality	· Evidence of validity (I heard about the collaborative clinics from the literature and online courses. I feel that it was in a good way. Pharmacist 2)	
	Relative Advantage	· Timely communication (Usually the relationship (with the physician) is quite good and smooth in the process of communication. Pharmacist 1)<br>· Complementarity of disciplines	
	Adaptability	· Individualized care service (I see that (the patient) has lost a lot of weight in a short period of time, probably because of his poor lifestyle. I asked them about their meals and gave them some dietary advice. Pharmacist 3)	
	Trialability	· High recognition of pilot study	
	Complexity		· Long-term follow-up (There has to be enough time to communicate with (the patient), and it's hard to adhere to if something else interferes. Pharmacist 5)<br>· Large number of patients (The number of patients is snowballing. Sometimes we can't keep up. Physician 4<br>Afterward, When I managed more patients, I feel overwhelmed. Pharmacist 2)<br>· Complexity of questionnaire (Some of the questions inside the questionnaire are not understandable to the patient and need to be explained to him. Pharmacist 5<br>Some patients have communication barriers because of their age. Pharmacist 10)
	Design Quality & Packaging	· Patient-centered collaborative care model (Pharmacists may not have enough influence, and it's definitely not enough for a pharmacist alone to manage a patient; they need to work with physicians. Pharmacist 12)	
	Cost		· Staffing and work time (We systematically train our staff to be qualified in the collaborative clinics or the pharmacy clinics. Pharmacist 3)<br>· Dedicated consulting room
Outer setting	Patient Needs & Resources	· Regular follow-up visits and reminders (The pharmacist's	· Poor health Literacy in primary areas (Our hospital is located in a

		reminders are very helpful. sometimes I don't pay attention to medication and they always remind. Patient 11)<br>· Pharmacy counseling (I ask the pharmacist if I should adjust my medication when my blood glucose gets high. Patient 12)<br>· Disease education (I instruct patients on blood sugar control precautions and lifestyle. Pharmacist 2)<br>· Medication guidance (I was helped in my knowledge of medication. Patient 22)<br>· Psychological support (Some patients feel that it is a bit of anxiety. Pharmacist 12<br>People with diabetes need psychological comfort and psychological adjustment. Patient 21)<br>· Medication cost-effectiveness (I would suggest a more economical drug in its class. Pharmacist 8)<br>· mHealth intervention (Manage all patients remotely and send messages through smartphone. Pharmacist 6)	remote area, so many patients have poor health concepts and need detailed communication. Pharmacist 8<br>Some patients have poor quality of health, which may be due to a lower level of education in the villages. Pharmacist 5)<br>· Distance or transportation difficulties (Some patients live in remote villages and it is not easy for them to come here. This is an objective factor. Pharmacist 6)<br>· Unfamiliar with smartphone (Older patients are less likely to use WeChat, and some may even have phones without Internet access. Pharmacist 6)<br>· Public stigma (Getting diabetes makes patients feel ashamed. Pharmacist 6)<br>· Insufficient communication with pharmacists (Patient were more likely to trust their physicians and communicate with them. Pharmacist 6)
	Cosmopolitanism	· Enhanced team-based management (Our endocrinologist, pharmacist, and health manager are all involved in the management of patients with diabetes. Pharmacist 6)<br>· online meeting (We have had several online meetings to share our experiences with other county hospitals. Pharmacist 9)	
	Peer Pressure	· Progress report (Progress is reported to the primary research center via online meetings. Pharmacist 9)<br>· Informal communication among units (Similar to Ningxiang and Pingjiang, we have a positive development. Pharmacist 3)	
	External Policy & Incentives	· Patient requirements<br>· Incentive policies from study<br>· Academic development (We are proactively communicating with patients, providing medication instructions and lifestyle guidance, and hope to gradually expand the influence of pharmacists. Pharmacist 3)<br>· Leadership support (The director of our hospital was a pharmacist, and he is very supportive and challenges us to be high quality. Pharmacist 8)	· Not included in performance appraisal (I spend extra time doing patient education because of the lack of incentives. Physician 2)<br>· Lack of charging mechanism (Pharmacy charge is a big deal. Pharmacist 3)
Inner setting	Structural Characteristics	· Clear delineation of workload (I have organized and requested their own duties. Pharmacist 3)	· Inappropriate staffing arrangements (There aren't as many pharmacists in the clinics, so staffing resources are important to consider. Physician

		4)
Networks & Communications	· Mature cultivating mode (When I first started in the profession, I was shadowing the director to the consultations. We have a proven model of teaching. Pharmacist 2)<br>· High quality teamwork (Our team shares the work and I have 15 patients to manage. Pharmacist 6)<br>· Reporting and discussion	· Lack of communication within the organization
Culture	· Collective learning (Our department conducts intra-departmental lectures as professional studies. I gave the first lecture. Pharmacist 2)<br>· Encouragement of capacity enhancement (The director said you have to improve your learning skills, and change your perceptions in the midst of continuous learning. Pharmacist 1)<br>· Patient-centered values (By improving our service, we are able to create a sense of warmth in the patient, so that they are willing to have follow-up visits and have a high level of compliance. Pharmacist 1)	
Tension for Change	· Paradigm shift in pharmacy services (Changing the way we work requires us to communicate with the clinical department and patients. Pharmacist 4)<br>· Irrational drug use in primary areas (Some medicines are not really necessary, but the situation is worse in primary areas. Pharmacist 3)<br>· Large patient population and disease burden (There are many people with diabetes, but it’s not well controlled. It's a big burden on the patient and hospital. Pharmacist 5)	
Compatibility	· Responsibility and values (We have aspiration for being good pharmacists. Pharmacist 1)	
Relative Priority	· Integration into daily work (I used to attend the collaborative clinics three times a week, now it's twice a week. Pharmacist 2)	· Priority for daily work (We're all in overtime right now. Pharmacist 12<br>Pharmacists used their spare time to follow up with patients, but they are already multi-tasking. Pharmacist 9)
Organizational Incentives & Rewards	· Leadership encouragement (I think our pharmacists are really doing well. Pharmacist 8)<br>· Partner recognition	
Learning Climate	· Positive learning (I follow diabetes on the Internet and also listen to courses in software such as DXY. Pharmacist 2)<br>· Acceptance of new things (We definitely support patients attending	

		collaborative clinics because the guidance is very helpful. I'm very supportive of emerging things. Physician 7)<br>· Reflection and improvement (I think it's much better now than at the beginning. It's important to keep improving, to gain experience and to summarize. Pharmacist 2)	
	Leadership Engagement	· Collective training and learning<br>· Leadership support and oversight (Our leaders are willing to implement, gave us the platform and opportunity to try. Pharmacist 2)<br>· Leaders assist with inter-section communication (I communicate with other department managers to create a better working environment for pharmacists. Pharmacist 3)	· Lack of leadership oversight (I'm embarrassed that I haven’t done much work as a leader. Pharmacist 8)
	Available Resources	· Dedicated office (We secured a dedicated site from the hospital. Pharmacist 3)<br>· Specialist pharmacists	· Inadequate personnel competencies (I always feel the urge to improve my professionalism. Pharmacist 2)<br>· Conflicting schedules (The lack of planning is the reason why the work is not efficient. I feel that my work is disorganized. Pharmacist 1)
	Access to Knowledge & Information	· Vocational education (At the very beginning of the program's training, I participated in the entire process. Pharmacists 10)<br>· Experience Analysis Sessions (I learn from other people's experiences through online meetings. Pharmacist 2)	
Characteristics of individuals	Knowledge & Beliefs about the Intervention	· Sense of accomplishment and honor (The high compliance of patients makes me feel honored and rewarded. Pharmacist 1)<br>· Acceptance of patients (Collaborative clinics could be used as a platform to let patients gradually recognize us. Pharmacist 1)	· Unsure convincing (It is not feasible to promote it in our hospital, which is specialized in oncology and is not the preference of diabetic patients. Pharmacist 9)
	Self-efficacy	· High professional competence (We are experts in medication use and could give professional guidance to patients. Pharmacist 3)<br>· High recognition of physicians (Pharmacists are able to share some of the physician's work and even refine it. Physician 8)<br>· Better patient outcomes (They (patients) like the collaborative clinic because it's very effective. Pharmacist 1)	· Resistance to implement (I haven't put in enough time or effort, and there’s more to learn. Pharmacist 1<br>I don't think I'm doing a perfect job because I also have a lot of daily work, so sometimes I don't follow up with patients timely. Pharmacist 6)
	Individual Stage of Change	· Career experience accumulation (Rich clinical experience could be able to help physicians. Right now, I’m just in the way of skill accumulation and	

		learning. Pharmacist 9)	
	Other Personal Attributes	· Internal drive (I think pharmacists are working hard. Pharmacist 3<br>I think they are very conscientious and active in implementation. Physician 5)<br>· Competence (It's competent and not very difficult if you apply yourself. Pharmacist 1)<br>· Responsibility (I think they (pharmacists) are very responsible. When patients come, they would make an appointment in advance. Physician 4)<br>· Learning ability	
Process	Planning	· Preliminary research<br>· Well-established plan (The preparation was good, so I went to the clinic without any problems. Pharmacist 2)<br>· Regular review meeting (We will be criticized for our failure to accomplish the task at the meeting. Pharmacist 8)	
	Engaging	· Executive Team<br>· Communication and Discussion	
	Executing		· COVID Outbreak (It seriously affects the trial process. Because some patients had found the process of care-seeking complicated, it’s even more so during the epidemic. Pharmacist 10)

Supplementary material 7 The ratings of discriminative constructs of CFIR between high and low implementation units

Domains	High implementation units		Low implementation units	Distinguishing constructs
	Unit 1(taoyuan)	Unit 2(liuyang)	Unit 3(huaihua)	
Patient Needs & Resources	+1<br>(I instruct patients on blood sugar control precautions and lifestyle. Pharmacist 2<br>When I managed more patients, I feel overwhelmed. Pharmacist 2<br>When I saw the diagnosis, I was very frustrated. But the physicians and pharmacists took good care of me and I am very grateful. Patient 8<br>With our enhanced services, patients are more compliant and willing to receive follow-up visit. pharmacist 1)	+1<br>(The pharmacist's reminders are very helpful. sometimes I don't pay attention to medication and they always remind. Patient 11<br>I ask the pharmacist if I should adjust my medication when my blood glucose gets high. Patient 12<br>Some patients have poor quality of health, which may be due to a lower level of education in the villages. Pharmacist 5<br>Some patients live in remote villages and it is not easy for them to come here. This is an objective factor. Pharmacist 6<br>Patient were more likely to trust their physicians and communicate with them. Pharmacist 6)	0<br>(Our hospital is located in a remote area, so many patients have poor health concepts and need detailed communication. Pharmacist 8<br>I was helped in my knowledge of medication. Patient 22<br>This clinic should be initiated by the physician and the patients will definitely trust it. Because patients think that pharmacists are in charge of dispensing and may feel distrustful of the patient management that you carry out. Patient 22<br>Because it's not convenient for me to get here and I have to pay for registration, I feel it's useless. I get my blood glucose tested for free at the pharmacy or at the village health center. -Patient 18)	Weak
Structural Characteristics	+1<br>(I have organized and requested their own duties. Pharmacist 3)	+1<br>(Six full-time clinical pharmacists and TDM pharmacists participate with a reasonable distribution of tasks.)	-2<br>(I left the project up to the executive leader. Pharmacist 8<br>She is too busy on her own, and has a lot of daily work. pharmacist 9)	Strong
Networks & Communications	+1<br>(When I first started in the profession, I was shadowing the director to the consultations. We have a proven model of teaching. Pharmacist 2)	+2<br>(Our team shares the work and I have 15 patients to manage. Pharmacist 6)	-2<br>(I took a vacation after 2-3 months, so I didn't get much done. Pharmacist 9<br>I didn't do much work, I just assisted the leader when she was busy. Pharmacist 11)	Strong
Relative Priority	+2<br>(I used to attend the collaborative clinics three times a week, now it's twice a week. Pharmacist 2)	+1<br>(We conducted several collaborative clinic visits with physicians at the beginning of the study. Pharmacist 5)	-1<br>(We're all in overtime right now. Pharmacist 12<br>Pharmacists used their spare time to follow up with patients, but they are already multi-tasking. Pharmacist 9)	Strong
Learning Climate	+2	+1	0	Strong

	(I follow diabetes on the Internet and also listen to courses in software such as DXY. Pharmacist 2<br>I think it's much better now than at the beginning. It's important to keep improving, to gain experience and to summarize. Pharmacist 2)	(We definitely support patients attending collaborative clinics because the guidance is very helpful. I'm very supportive of emerging things. Physician 7)		
Leadership Engagement	+2<br>(There is no opportunity for pharmacists to demonstrate professional competence in other county hospitals. Pharmacist 1<br>Our leaders are willing to implement, gave us the platform and opportunity to try. Pharmacist 2<br>I communicate with other department managers to create a better working environment for pharmacists. Pharmacist 3)	+1<br>(Leaders mobilized all clinical pharmacists to participate.)	-1<br>(I'm embarrassed that I haven’t done much work as a leader. Pharmacist 8<br>The workload was assigned to the younger pharmacists. Pharmacist 10)	Strong
